# Characteristics, Management, and Outcomes of Community-Acquired Pneumonia due to Respiratory Syncytial Virus: A Retrospective Study

**DOI:** 10.1155/2023/4310418

**Published:** 2023-03-06

**Authors:** Ibrahim Bahabri, Abdulaziz Abdulaal, Thamer Alanazi, Sultan Alenazy, Yasser Alrumih, Rakan Alqahtani, Sameera Al Johani, Mohammad Bosaeed, Hasan M. Al-Dorzi

**Affiliations:** College of Medicine-Riyadh, King Saud bin Abdulaziz University for Health Sciences, King Abdullah International Medical Research Center, Department of Medicine, King Abdulaziz Medical City, Ministry of National Guard Health Affairs, Riyadh, Saudi Arabia

## Abstract

**Background:**

Respiratory syncytial virus (RSV), a well-known cause of bronchiolitis in children, can cause community-acquired pneumonia (CAP) in adults, but this condition is not well studied. Hence, we described the characteristics and outcomes of patients hospitalized for CAP due to RSV.

**Methods:**

This was a retrospective study of patients admitted to a tertiary-care hospital between 2016 and 2019 with CAP due to RSV diagnosed by a respiratory multiplex PCR within 48 hours of admission. We compared patients who required ICU admission to those who did not.

**Results:**

Eighty adult patients were hospitalized with CAP due to RSV (median age 69.0 years, hypertension 65.0%, diabetes 58.8%, chronic respiratory disease 52.5%, and immunosuppression 17.5%); 19 (23.8%) patients required ICU admission. The median pneumonia severity index score was 120.5 (140.0 for ICU and 102.0 for non-ICU patients; *p* = 0.09). Bacterial coinfection was rare (10.0%). Patients who required ICU admission had more hypotension (systolic blood pressure < 90 mmHg) and a higher prevalence of bilateral infiltrates on chest X-ray (CXR) (89.5% versus 32.7%; *p* < 0.001). Systemic corticosteroids were used in 57.3% of patients (median initial dose was 40 mg of prednisone equivalent) with ICU patients receiving a higher dose compared to non-ICU patients (*p* = 0.02). Most (68.4%) ICU patients received mechanical ventilation (median duration of 4 days). The overall hospital mortality was 8.8% (higher for ICU patients: 31.6% versus 1.6%, *p* < 0.001).

**Conclusions:**

Most patients with CAP due to RSV were elderly and had significant comorbidities. ICU admission was required in almost one in four patients and was associated with higher mortality.

## 1. Introduction

Community-acquired pneumonia (CAP) continues to pose a significant burden on healthcare systems in terms of morbidity and mortality [1, 2]. Viruses have been underrecognized as a cause of CAP, with perhaps influenza being a prominent exception [3]. Newer molecular techniques, including the polymerase chain reaction (PCR), have changed this concept. In one study of 198 samples from the respiratory tract of patients with CAP, PCR detected viruses in nearly a quarter of all cases [4]. The most commonly detected viruses were influenza A and adenovirus [4]. A study of pooled cohorts of CAP patients found that viruses were detected in 22% of cases, rising to 29% in studies where PCR was performed [5]. The most common viral pathogens identified in the review were influenza viruses (9%), followed by human rhinovirus (5%) [5]. Respiratory syncytial virus (RSV), an enveloped, nonsegmented, and ssRNA virus that belongs to the *Paramyxoviridae* family, has not been well established as a cause of CAP in adults with two studies finding RSV in 2% and 2.5% of overall cases when PCR was performed [4, 5].

RSV is a well-known cause of bronchiolitis in children [6], with a well-known seasonal outbreak pattern leading to endemics between November and April in the northern hemisphere (late autumn to early spring) [7–9]. A US-based study of over 5000 children with lower respiratory tract infections found that RSV was detected in 18% of the total cases and accounted for 20% of hospitalizations [7]. RSV bronchiolitis is associated with a low mortality rate of 0-0.3% as observed in multiple studies [7–9]. Another spectrum of RSV-related disease is seen in adults, where RSV causes an influenza-like illness [10, 11], with two prominent distinctions between RSV and influenza being the higher frequency of rhinorrhea and the higher likelihood of wheezing (including patients with no history of chronic airway disease) and receiving therapy for bronchospasm in RSV cases [12–14]. RSV has also been implicated in chronic obstructive pulmonary disease exacerbations [15, 16] and severe pneumonia [17, 18]. Elderly populations seem to be at particular risk of developing severe RSV pneumonia. A study of 149 nursing home residents with acute lower respiratory tract infections found that RSV was detected in 62 patients and was associated with a significantly more severe disease compared to rhinovirus [19]. Another study of an RSV outbreak in 40 out of 101 nursing care facility residents showed a mortality rate of 20% [20]. CAP due to RSV can also occur in noninstitutionalized adults [13, 14]. Another group at risk of RSV pneumonia is the immunocompromised patients, especially bone marrow transplant recipients [17, 18, 21, 22]. Treatment is primarily supportive, but usage of ribavirin in some of the immunocompromised patients has demonstrated some clinical improvement, especially with early implementation [18]. RSV infection may be associated with higher mortality compared to influenza [13].

Data on pneumonia caused by viruses other than Middle East respiratory syndrome coronavirus and severe acute respiratory syndrome coronavirus 2 in Middle Eastern populations are scarce [23, 24]. A study on patients with viral pneumonia in a tertiary-care hospital in Riyadh found that the most commonly identified virus was influenza A (non-H1N1)/influenza B (216 patients), followed by H1N1 influenza (150 patients), and Middle East respiratory syndrome coronavirus (82 patients) [23]. In this study, we aimed to characterize patients admitted with CAP due to RSV and report their outcomes.

## 2. Methods

### 2.1. Patients and Settings

This was a retrospective cohort study of adult patients who had CAP due to RSV and were hospitalized in a 1400-bed tertiary-care referral hospital in Riyadh, Saudi Arabia, between January 1, 2016, and December 31, 2019. Patients aged 14 and older with clinically diagnosed CAP and a positive respiratory PCR sample for RSV within 48 hours of admission were included in our study. Our laboratory used BioFire FilmArray pouch (BioFire™ Diagnostics, Inc., Salt Lake City, UT, USA) for the respiratory multiplex PCR assay. It stored all the necessary reagents for sample preparation, reverse transcription PCR, and detection in a freeze-dried format. In this study, accepted respiratory samples were sputum, endotracheal aspirate, and bronchoalveolar lavage. During a test run, the BioFire System extracted and purified all nucleic acids from the unprocessed sample. Next, it performed nested multiplex PCR in two stages. The first stage included a single, large-volume, multiplexed reaction. The second stage included individual, single-plex reactions to detect the products from the first stage. Using endpoint melting curve data, BioFire System software automatically analyzed the results for each target on the panel. When the run was complete, the software reported whether each pathogen was detected in the sample.

### 2.2. Data Collection

The list of patients who tested positive for RSV within the study period was obtained from the microbiology laboratory of the hospital. We collected the following data: demographics, comorbid conditions, month of hospitalization, signs and symptoms documented in the medical records, and pertinent laboratory results and radiographic findings at presentation. We also calculated the pneumonia severity index (PSI) [25] and noted the provided management (use of antimicrobials and antivirals, steroid use and dosage, ICU admission, intubation, mechanical ventilation, and the use of vasopressors). We also noted the rate of bacterial coinfection and superinfection (positive bacterial sputum culture within 48 hours in cases of coinfection and more than 48 hours in cases of superinfection). The primary outcome of our study was in-hospital mortality. Secondary outcomes included ICU mortality, duration of mechanical ventilation, tracheostomy, length of stay in the ICU and hospital, and hospital readmission within 30 days.

### 2.3. Statistical Analysis

The patients were divided into two main groups based on whether they required ICU admission or not. Continuous variables were presented as median with interquartile range (IQR) and compared using either Student's *t*-test or Mann–Whitney *U*-test, depending on the normality of distribution. Categorical variables were presented as frequency with percentage and compared using either the chi-square test or Fisher's exact test, as appropriate. The hospital mortality was compared between clinically important subgroups of patients: age ≤ 65 versus >65 years, PSI ≤ 90 (class I-III, which usually indicates low risk of mortality) versus >90 (class IV-V, which usually indicates moderate-high risk of mortality) [25], immunocompromised versus not immunocompromised, corticosteroid versus no corticosteroid use, ICU admission versus no ICU admission, vasopressor therapy versus no vasopressor therapy, and mechanical ventilation versus no mechanical ventilation. All statistical tests were considered significant at a *p* value less than 0.05. Statistical analysis was performed using SPSS (SPSS Inc., SPSS for Windows, version 16.0, Chicago, IL, SPSS Inc.).

## 3. Results

### 3.1. Baseline Characteristics and Presenting Symptoms and Signs

**Figure 1:**
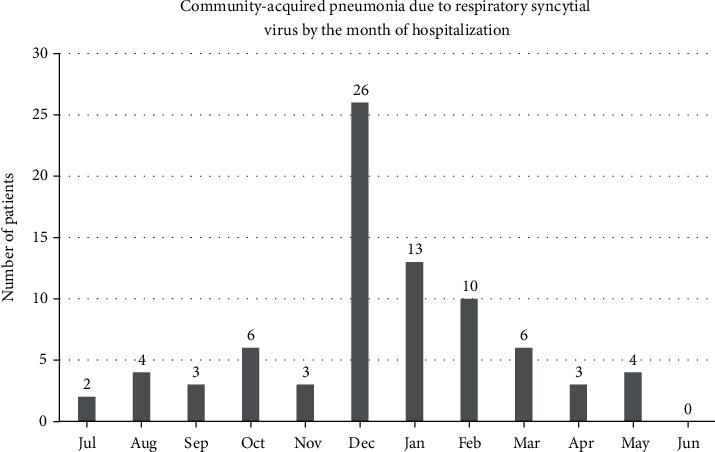
Distribution of hospital admissions for community-acquired pneumonia due to respiratory syncytial virus in the different months of the year.

Eighty adult patients were hospitalized with CAP due to RSV during the study period.  Figure 1 describes the number of hospital admissions by the month of the year. It shows that most (49/80, 61.3%) admissions were from December to February. The baseline data of the study patients are presented in Table 1. The median age of patients was 69 years (IQR: 57.5, 79.7), typically with multiple comorbidities. The most common comorbidities were hypertension (65%) and diabetes (58.8%), followed closely by chronic respiratory disease (52.5%) and heart failure (38.8%). Fourteen (17.5%) patients were immunosuppressed: 8 patients (10% of the overall cohort) received solid organ transplantation (6 had kidney transplant and 2 liver transplant) and were on a combination of tacrolimus (*N* = 5), corticosteroids (*N* = 8), mycophenolate (*N* = 5), or cyclosporin (*N* = 3); 3 patients had hematologic disorders; and 3 were on immunosuppressive medications for tuberculous meningoencephalitis (*N* = 1), systemic lupus erythematosus with nephritis (*N* = 1), and interstitial lung disease (*N* = 1). Sixteen patients (20%) were bedbound at baseline prior to presentation. The most common presenting symptom was shortness of breath (88.8%), followed by productive cough (72.5%). Only 47.5% of patients had fever reported from history or documented on presentation.

Nineteen (23.8%) patients required ICU admission. No significant differences were found in the demographics, comorbidities including the presence of immunosuppression, mean PSI, and the baseline laboratory findings of ICU patients compared to non-ICU patients (Table 1). ICU patients were significantly more likely to have bilateral interstitial infiltrates on chest X-ray compared to non-ICU patients (89.5% versus 32.7%, *p* < 0.001) and bilateral pleural effusions (47.4% versus 14.5%, *p* = 0.009).

Only one patient (1.25%) had RSV and influenza virus coinfection. Bacterial coinfection was found in five non-ICU patients (four cases had *Staphylococcus aureus* where two were methicillin-resistant and one had both *Klebsiella pneumoniae* and *Pseudomonas aeruginosa*) and three ICU patients (two *Streptococcus pneumoniae* and one *Klebsiella pneumoniae*).

### 3.2. Management

Management of the study patients is presented in Table 2. Sixty-one (76.3%) patients received empiric oseltamivir (no significant difference between ICU and non-ICU patients and between immunosuppressed and immunocompetent patients). Oral ribavirin was provided to one patient who was immunosuppressed after RSV infection was diagnosed. Most patients in our cohort were started on empiric antibiotics (96.2%). A combination of ceftriaxone and a macrolide was the most commonly prescribed initial regimen (40.1%), followed by piperacillin-tazobactam and a macrolide (37.6%). The use of empiric piperacillin-tazobactam or meropenem in combination with a macrolide was similar in patients who were immunosuppressed and those who were not (4/14 patients (28.6%) versus 19/66 (28.8%), respectively; *p* = 1.0). On the other hand, patients with bacterial coinfection were more likely to receive piperacillin-tazobactam or meropenem in combination with a macrolide compared to those without bacterial coinfection (5/8 patients (62.5%) versus 18/72 patients (25.0%), respectively; *p* = 0.04). In general, there were no significant differences in the empiric antibiotics between ICU and non-ICU patients. More than half (53.7%) of the study patients received corticosteroids as part of initial therapy, and although there was no significant difference in the rate of steroid use in ICU patients compared to non-ICU patients, ICU patients were started on a higher dose (median of 55 mg of prednisone or its equivalent) compared to non-ICU patients (median of 40 mg of prednisone or its equivalent, *p* = 0.02).

### 3.3. Outcomes of Patients

Table 3 describes the outcomes of the study patients. The overall hospital mortality in the study cohort was 8.8% with a significantly higher mortality rate in ICU patients (31.6% versus 1.6%, *p* < 0.001). The 30-day readmission rate was 24.7% with no significant difference between ICU and non-ICU patients.

**Figure 2:**
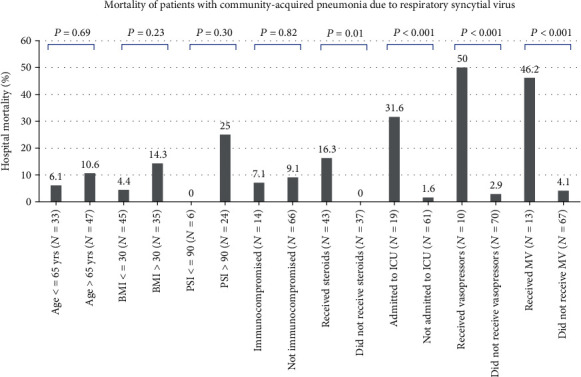
Hospital mortality in clinically relevant subgroups of patients who had community-acquired pneumonia due to respiratory syncytial virus. BMI: body mass index; ICU: intensive care unit; PSI: pneumonia severity index; MV: mechanical ventilation.

Figure 2 describes the hospital mortality in clinically important subgroups of patients. There were no significant differences in the mortality rates between older and younger patients and between immunosuppressed and immunocompetent patients. Patients who received corticosteroids and those who were admitted to the ICU and had organ support had higher mortality rates than those who did not.

## 4. Discussion

The main findings of this study were the following: most cases of CAP due to RSV in adults requiring hospitalization occurred during the colder months in Riyadh, Saudi Arabia (December to February); most patients were elderly with multiple comorbidities; almost one-fourth of hospitalized patients were admitted to the ICU; and almost 1 in 10 patients died in the hospital with most deaths in patients requiring ICU admission and organ support.

The median age in our cohort was 69 years, which conforms with the existing data showing that elderly patients aged ≥65 years represent a high proportion of hospitalized adults with RSV infection [13, 14]. Twenty percent of our study population was bedbound at baseline. These patients are probably at increased risk for viral infections and may correspond to people staying at nursing homes in other countries [19, 20]. Hypertension (65.0%), diabetes (58.8%), and chronic respiratory disease (52.5%) were especially prevalent among our study population, emphasizing that these comorbidities might be significant risk factors for RSV pneumonia requiring hospitalization as observed in previous studies on patients with RSV pneumonia [13, 14]. Chronic immunosuppression, although present in 14/80 patients (17.5%), was not significantly different between ICU and non-ICU patients. Only 1/8 patients (12.5%) with a history of an organ transplant required admission to the ICU; this is much lower than the rates of ICU admissions of transplant patients in multiple studies [18, 21]. The median PSI was 120.5 overall (102.0 for non-ICU patients vs. 140.0 for ICU patients); the difference in the PSI was not statistically significant (*p* = 0.09).

The majority of patients presented with shortness of breath (88.8%) and cough (96.2%), which was more commonly productive than dry (58 patients versus 19 patients); these findings are consistent with previous data regarding RSV pneumonia [11–14]. This prevalence of productive cough cannot be fully attributed to a concomitant bacterial organism as only 10% of all patients had a bacterial coinfection detected by respiratory culture, a lower rate of bacterial coinfection compared to other studies [13, 16, 26]. Five (6.3%) patients had hemoptysis, but only 1 patient required ICU admission. The classic signs of systemic viral illness such as myalgias/arthralgias (20%) and headache (7.5%) were not found in the majority of our patients and were reported at a similar frequency in the study by Mathur et al. [12]. Less than half of the patients (47.5%) were febrile on admission, with an even lower rate of fever on presentation among ICU patients (36.8%), indicating that a lack of fever was insufficient to exclude CAP due to RSV or to even indicate less disease severity. This is lower than the findings of Dowell et al. where fever was reported in 61% of patients [14]. In our study, bilateral lung infiltrates and pleural effusions were common, similar to the findings of other studies [17, 18, 21].

In the current study, most patients (76.3%) received empiric oseltamivir with a similar rate among ICU and non-ICU patients. The use of empiric oseltamivir is common in clinical practices [27], especially since most of the study patients were admitted in the winter time, which is the peak time for influenza infection in Saudi Arabia [28]. Only one patient who was immunosuppressed received oral ribavirin. Although oral or nebulized ribavirin has been used for the treatment of severe RSV infections in immunosuppressed adult patients with observed benefits, most studies on the effectiveness of ribavirin were uncontrolled and not adequately powered [29]. Most of the study patients received antibiotics within the first 24 hours of admission, with antipseudomonal beta-lactams being commonly used. No significant difference was found in the usage of antibiotics between ICU and non-ICU patients and between immunosuppressed and immunocompetent patients. Broad-spectrum antibiotics are frequently used in patients with viral pneumonia or other respiratory tract infections [26, 30, 31]. These findings indicate the need for reliable methods that distinguish early viral from bacterial infection in patients with pneumonia and for antibiotic and antiviral stewardship where empirical treatment would be adjusted based on the result of the investigations [32]. Systemic corticosteroids were commonly (57.3% of patients) used with a median initial dose of 40 mg of prednisone or its equivalent. This could be because chronic respiratory diseases were prevalent in the study patients. Some of our patients may have exhibited wheezing leading to the common use of systemic corticosteroids.

Patients with CAP due to RSV had considerable morbidity and mortality in our study. Nearly one in four patients (23.8%) required ICU admission, which is in line with the 21-25% ICU admission rate in other studies [13, 14]. The overall mortality in our study was 8.8%, which is similar to the 10% mortality rate found by Falsey et al. and Yoon et al. [13, 22] but is considerably lower than the rates in studies in immunocompromised patients where mortality ranged from 36 to 50% [17, 18]. Naturally, mortality was significantly higher in ICU patients compared to non-ICU patients (31.6% versus 1.6%, *p* < 0.001). Significantly different variables between ICU and non-ICU patients included the presence of bilateral chest infiltrates on chest imaging (89.5% for ICU patients versus 32.7% for non-ICU patients; *p* < 0.001). Thirteen out of 80 (16.3%) patients in our cohort required mechanical ventilation. This was higher than the rate observed by Falsey et al. [13] but considerably lower than that of the study by Hertz et al. in immunocompromised patients where 4 out of 11 (36%) required mechanical ventilation [18]. We have observed higher mortality in patients who received corticosteroids. This observation may be explained by the fact that patients who received corticosteroids were sicker.

The main limitation of our study is its retrospective nature, making it difficult to establish causation between characteristics, exposures, and outcomes. The sample size, even though being relatively large compared to that of other studies, prevented the performance of reliable multivariable logistic regression analyses to assess the risk factors for ICU admission and mortality. Being a single-center study also limits the generalizability of our findings. The study also focused on patients hospitalized for CAP where outpatient cases were not accounted for.

In conclusion, most patients with CAP due to RSV were elderly with significant comorbidities. ICU admission was required in almost one in four patients and mechanical ventilation in almost one in six patients. The overall hospital mortality was 8.8% with patients who were admitted to the ICU having significantly higher mortality approaching 1 in 3 patients.

## Figures and Tables

**Table 1:** Characteristics of patients admitted with community-acquired pneumonia due to respiratory syncytial virus.

Variable	All patients *N* = 80	ICU admission *N* = 19	No ICU admission *N* = 61	*p* value
Age (years), median (IQR)	69.0 (57.5, 79.7)	71.0 (57.0, 81.0)	66.0 (58.0, 79.0)	0.534
Male sex, *N* (%)	41 (51.3)	13 (68.4)	28 (45.9)	0.09
BMI (kg/m^2^), median (IQR)	29.0 (24.0, 36.8)	31.2 (23.3, 38.5)	29.0 (24.0, 36.8)	0.874
Smoking, *N* (%)	8 (10.0)	3 (15.8)	5 (8.2)	0.335
Comorbidities, *N* (%)				
Diabetes	47 (58.8)	10 (52.6)	37 (60.7)	0.535
Hypertension	52 (65.0)	12 (63.2)	40 (56.6)	0.847
Hypothyroidism	13 (16.3)	2 (10.5)	11 (18.0)	0.439
Heart failure	31 (38.8)	5 (26.3)	26 (42.6)	0.203
Stroke	12 (15.0)	2 (10.5)	10 (16.4)	0.532
Chronic kidney disease	19 (23.8)	5 (26.3)	14 (23.0)	0.763
Dialysis	5 (6.3)	2 (10.5)	3 (4.9)	0.378
Intermittent dialysis	21 (26.3)	3 (15.8)	18 (29.5)	0.235
Dyslipidemia	28 (35.0)	5 (26.3)	23 (37.7)	0.363
Chronic respiratory disease	42 (52.5)	9 (47.4)	33 (54.1)	0.608
Liver disease	5 (6.3)	1 (5.3)	4 (6.6)	0.839
Immunosuppression	14 (17.5)	4 (21.1)	10 (16.4)	0.641
Malignancy	4 (5.0)	3 (15.8)	1 (1.6)	0.013
Transplant	8 (10.0)	1 (5.3)	7 (11.5)	0.431
Bedbound, *N* (%)	16 (20.0)	4 (21.1)	12 (19.7)	0.895
Presenting symptoms, *N* (%)				
Fever	38 (47.5)	7 (36.8)	31 (50.8)	0.287
RR > 30/min	26 (32.5)	9 (47.4)	17 (27.9)	0.113
Systolic < 90 mmHg	9 (11.3)	5 (26.3)	4 (6.6)	0.017
*T* < 35 or >39.9°C	12 (15.0)	3 (15.8)	9 (14.8)	0.912
Pulse > 125/min	17 (21.3)	6 (31.6)	11 (18.0)	0.208
BUN > 11 mmol/L	29 (36.3)	7 (36.8)	22 (36.1)	0.951
Dyspnea	71 (88.8)	16 (84.2)	55 (90.2)	0.473
Dry cough	19 (23.8)	5 (26.3)	14 (23.0)	0.763
Purulent cough	58 (72.5)	12 (63.2)	46 (75.4)	0.296
Chest pain	8 (10.0)	0 (0.0)	8 (13.1)	0.096
Myalgia/arthralgia	16 (20.0)	4 (21.1)	12 (19.7)	0.895
Fatigue	21 (26.3)	6 (31.6)	15 (24.6)	0.545
Headache	6 (7.5)	3 (15.8)	3 (4.9)	0.116
Hemoptysis	5 (6.3)	1 (5.3)	4 (6.6)	0.839
Pneumonia severity index, median (IQR)	120.5 (94.0, 159.5)	140.0 (97.0, 17.1)	102.0 (79.0, 145.0)	0.085
Laboratory findings, median (IQR)				
White blood cell (10^9^/L)	8.9 (5.8, 12.3)	7.5 (4.7, 13.5)	9.1 (6.3, 12.1)	0.619
Neutrophil (%)	70.9 (58.0, 80.5)	74.0 (63.4, 86.5)	69.6 (50.8, 79.8)	0.068
Lymphocyte (%)	14.8 (8.4, 20.7)	11.4 (2.8, 19.9)	14.9 (9.5, 20.9)	0.239
Hematocrit	0.39 (0.34, 0.44)	0.38 (0.29, 0.44)	0.39 (0.34, 0.44)	0.475
PTT (seconds)	29.5 (26.8, 33.9)	30.45 (28.3, 33.6)	28.5 (26.6, 34.0)	0.253
INR	1.16 (1.05, 1.34)	1.18 (1.13, 1.27)	1.15 (1.04, 1.36)	0.571
Sodium (meq/L)	136.0 (133.0, 139.0)	136.0 (129.0, 142.0)	136 (133, 139)	0.793
Lactic acid (mmol/L)	1.86 (1.26, 3.06)	2.48 (1.2, 3.0)	1.78 (1.26, 2.74)	0.405
Creatinine (*μ*mol/L)	86.5 (64.5, 133.75)	96.0 (68.0, 163.0)	82 (63.5, 129.5)	0.553
pH	7.37 (7.30, 7.41)	7.37 (7.22, 7.41)	7.36 (7.33, 7.41)	0.534
PaO_2_ (mmHg)	63.1 (52.0, 76.0)	64.4 (61.4, 81.3)	62.9 (48.2, 73.4)	0.333
Chest X-ray findings				
Unilateral infiltrates	20/74 (27.0)	1/19 (5.3)	19/55 (34.5)	0.015
Bilateral infiltrates	35/74 (47.3)	17/19 (89.5)	18/55 (32.7)	<0.001
Unilateral pleural effusion	13/74 (17.6)	3/19 (15.8)	10/55 (18.2)	1.0
Bilateral pleural effusion	17/74 (23.0)	9/19 (47.4)	8/55 (14.5)	0.009
Chest computed tomography findings				
Unilateral infiltrates	8/26 (30.8)	1/5 (20.0)	7/21 (33.3)	1.0
Bilateral infiltrates	7/26 (26.9)	2/5 (40.0)	5/21 (23.8)	0.588
Unilateral pleural effusion	5/26 (19.2)	0/5 (0)	5/21 (23.8)	0.545
Bilateral pleural effusion	7/26 (26.9)	2/5 (40.0)	5/21 (23.8)	0.588

BMI: body mass index; PTT: partial thromboplastin time; INR: international normalized ratio; IQR: interquartile range; MV: mechanical ventilation.

**Table 2:** Management of patients admitted with community-acquired pneumonia due to respiratory syncytial virus.

Variable	All patients *N* = 80	ICU admission *N* = 19	No ICU admission *N* = 61	*p* value
Mechanical ventilation, *N* (%)	13 (16.2)	13 (68.4)	0 (0)	<0.001
Use of steroids, *N* (%)	43 (53.7)	14 (73.7)	29 (47.5)	0.065
Initial daily dose (in mg prednisone equivalent), median (IQR)	40.0 (32.5, 75.0)	55.0 (36.3, 100.0)	40.0 (30.0, 40.0)	0.02
Antimicrobial therapy, *N* (%)				
No initial antibiotic	3 (3.8)	1 (5.3)	2 (3.3)	0.562
Ceftriaxone ± macrolide	32 (40.1)	5 (26.3)	27 (44.4)	0.190
Piperacillin-tazobactam ± macrolide	30 (37.6)	9 (47.4)	21 (34.5)	0.416
Meropenem ± macrolide	3 (3.8)	2 (10.6)	1 (1.6)	0.139
Oseltamivir, *N* (%)	61 (76.3)	15 (78.9)	46 (75.4)	0.752

IQR: interquartile range.

**Table 3:** Outcomes of patients admitted with community-acquired pneumonia due to respiratory syncytial virus.

Variable	All patients *N* = 80	ICU admission *N* = 19	No ICU admission *N* = 61	*p* value
MV duration (days), median (IQR)	NA	4.0 (3.5, 30.0)	NA	NA
LOS in ICU (days), median (IQR)	NA	9.0 (5.0, 26.0)	NA	NA
LOS in hospital (days), median (IQR)	7.0 (4.0, 11.8)	12.0 (10.0, 31.0)	6.0 (4.0, 10.0)	<0.001
Tracheostomy, *N* (%)	3 (3.8)	3 (15.8)	0 (0)	0.02
Hospital mortality (%)	7 (8.8)	6 (31.6)	1 (1.6)	<0.001
30-day readmission	19 (24.7)	4 (25)	15 (24.6)	0.937

ICU: intensive care unit; IQR: interquartile range; LOS: length of stay.

## Data Availability

Data are available on reasonable request from the corresponding author.
